# The Treatment of Diabetes

**Published:** 1889-04

**Authors:** 


### EDITORIAL.

### *THE TREATMENT OF DIABETES.*

One of the most notable and valuable papers that has been presented to the Chicago Medical Society for some time, was one on "The Treatment of Glycosuria," by Dr. Charles W. Purdy. Beginning with the usual classification of diabetes under the two forms, of the milder manifestation of the disease, in which only small amounts of sugar appear in the urine, while the patient's health suffers little or no disturbance, and the more severe type, characterized by excessively saccharine urine, great thirst, polyuria, etc., leading more or less rapidly to extreme emaciation and death, Dr. Purdy goes on to point out some of the facts in regard to the glycogenic function of the liver, as taught by physiological chemistry.

The basis of the treatment of diabetes, in the opinion of Dr. Purdy, as well as of Dr. Pavy and other therapeutists, is diet. The foods that can be transformed into sugar must be prohibited. The hydrocarbons are of course the chief sugar-forming foods, and the more completely we can eliminate them from the food-supply in diabetes, the more completely will we be able to bring and hold the disease under control. First in importance is the question of bread. In regard to the so-called diabetic flours, breads, and cakes, Dr. Purdy does not hesitate to say that all these preparations now on the market are loaded with starch; are "a delusion and a snare." "Most samples of gluten flour," he says, "from which the starch is claimed to have been eliminated—or nearly so—contain from 20 to 40 per cent. of starch. I saw in Dr. Pavy's laboratory in London a few months since an analysis of one of the so-called diabetic flours on sale in our markets, which showed the starch contents to be nearly 60 per cent. Long before I became aware of these facts I found that I could not control typical cases of diabetes if I permitted the use of commercial flours so-called 'diabetic.' I need scarcely add that with the above figures before me I have discarded them altogether.

"The withdrawal of bread from the diet usually constitutes the most serious deprivation the diabetic patient has to encounter, although the appetite for bread is more largely a matter of taste and habit than of necessity. Some patients become quite reconciled to the change after a few weeks, and do not mind it, but usually the craving for bread of some kind remains more or less strong, and will not be supplanted by the use of other foods. In the latter class of cases, if strict dieting be demanded, I permit the moderate use of bread made from almond flour as first practiced, I believe, by Dr. Pavy. The almond is absolutely free from starch, but contains about 6 per cent. of sugar. The latter may be eliminated by boiling the meal in acidulated water for an hour or so and then straining it. The almond meal is not on sale in the markets; the large percentage of its contained oil (50 per cent.) renders it unfit for keeping sufficiently long for commercial purposes. In my own practice I direct the meal to be made as required by means of mills especially constructed for the purpose. Almond flour, when beaten up with eggs, may be raised with the aid of a little baking powder, and baked in small tins in an oven, and the resulting bread is relished by most of my patients as equally palatable with ordinary bread. It should be borne in mind that almond bread, as indeed all substitutes for common bread, should be used in moderation; otherwise patients deprived of other luxuries of food fly to the permitted bread with an avidity seemingly born of the thought that it is indeed the 'staff of life' instead of merely a substitute therefor. To make a substituted article of diet go further than the original one is more than is to be expected, even in these practical days, and yet I am led to believe that the failure in accomplishing this in the case of almond bread has led to its unjust condemnation by some in these cases."

Next in importance to the question of bread is that of the use of milk in diabetes. Dr. Purdy, after extensive trials of this article, has come to the conclusion that milk is successful chiefly, perhaps only, in milder forms of diabetes, of reflex origin. Such cases are controllable by moderate limitations of diet. In the more severe types of the disease Dr. Purdy has found that when the diet was rigidly restricted, save in the use of milk, the total exclusion of this article, without other change, caused a prompt reduction in the amount of sugar excreted, and often its total disappearance.

"Milk contains a very considerable amount of sugar (lactine), about half an ounce to each pint, and Dr. Pavy observes that this animal hydrocarbon 'comports itself in the intestinal canal precisely as does grape-sugar.' There can be little doubt, therefore, that in the more pronounced type of diabetes requiring a strict diet, milk should be excluded from the list. There is a form of glycosuria that occurs in obese and over-nourished subjects, in which the amount of sugar in the urine is usually small, and probably largely due to the ingestion of more hydrocarbons than the system is able to appropriate. Such cases are benefited, and indeed often cured, by a course of fasting. The 'milk cure' consisting of the exclusive use of skimmed milk is likely to benefit such cases because it is, in fact, a system of starving. Skimmed milk alone is not sufficient to long maintain proper nourishment to the system. In pronounced diabetes of central origin, where the assimilative powers of the system are weakened, and more or less emaciation has already set in, it would, therefore, seem absolute folly to confine the patient to skimmed milk, for under such circumstances death from inanition must be but a question of a short time. It is important to bear in mind that lactine is confined to the whey, and consequently the other derivatives of milk—as cheese, cream, curds and butter—are unobjectionable."

After reviewing the food-products applicable in diabetes, Dr. Purdy gives the foling *strict diabetic diet*, as appropriate in the more severe type of true diabetes of central origin:

"Meats of all kinds except livers; beef roasted, broiled, dried, smoked, cured, potted, or preserved in any way except with honey, sugar, or prohibited vegetables. Mutton, ham, tongue, bacon, sausages. Poultry and game of all kinds. Soups made from meats, without flour or prohibited vegetables. Eggs, butter, cheese, pure cream, curds, oil, gelatine and unsweetened jellies. Fish of all kinds except oysters and the inner parts of crabs and lobsters. Bread, biscuits, and cakes made from almond flour. Spinach, lettuce, olives, cucumbers, mushrooms, water-cresses, green cabbage. Almonds, walnuts, Brazil nuts, filberts, butternuts, cocoanuts. Salt, vinegar and pepper.

"*Drinks*, tea and coffee, mineral waters, whisky, gin and brandy, in moderation. Claret and Rhine wine.

"In mild forms of glycosuria some additions may be safely made to the above diet, and often with advantage. Since in such cases the sugar-forming powers of the organism are weaker; or, in other words, the assimilative powers for sugar and starch are greater, it is only necessary to limit, *not to curtail* the hydrocarbons. It seems necessary, therefore, to have at hand to draw upon a supplementary list of foods, which contain but limited amounts of these agents. The selection from the supplementary list should always be made with care; indeed, it should be almost as much a matter of experiment as rule, since we encounter wide differences in individual cases. Thus levulose—fruit sugar—is often well assimilated in the milder form of the disease, and this permits the inclusion of certain fruits in the supplementary list."

Following is the *supplementary diet:* "Cabbage, celery, radishes, cauliflower, green string beans, coldslaw, kraut, young onions, tomatoes, cranberries, apples if not sweet, milk in moderate quantities, and bran bread or gluten bread well toasted."

Saccharine should be used as a sweetening agent; it is entirely harmless.

Scarcely less important than the diet itself is the method of dieting. When the patient is placed upon diet, medicines should be excluded until the sugar excretion is reduced as much as possible by diet alone. The more objectionable foods should be cut off step by step, until sugar ceases to appear in the urine, or until an almost or absolutely exclusive animal diet is reached. The method of dieting, and the various steps, given by Dr. Purdy cannot be even outlined in the space at our command; the subject is so important that his whole article, which appears in the *Journal of the American Medical Association* of March 30, should be carefully read.

In regard to drugs, it may be said that there is very little evidence in favor of any one being entitled to confidence; they are probably nearly inert, so far as their influence over diabetes is concerned, when their effects are carefully discriminated from those produced by dieting. The legitimate field of drugs in diabetes is practically narrowed down to the treatment of its accompanying symptoms, such as disordered digestion, constipation, nervous troubles, etc.

